# Genome sequence and comparative analysis of a putative entomopathogenic *Serratia* isolated from *Caenorhabditis briggsae*

**DOI:** 10.1186/s12864-015-1697-8

**Published:** 2015-07-18

**Authors:** Feseha Abebe-Akele, Louis S. Tisa, Vaughn S. Cooper, Philip J. Hatcher, Eyualem Abebe, W. Kelley Thomas

**Affiliations:** Department of Molecular, Cellular, and Biomedical Sciences, University of New Hampshire, Durham, NH USA; Department of Computer Science, University of New Hampshire, Durham, NH USA; Department of Biology, Elizabeth City State University, 1704 Weeksville Road, Jenkins Science Center 421, Elizabeth City, NC 27909 USA; Hubbard Center for Genome Studies, 444 Gregg Hall, University of New Hampshire, 35 Colovos Road, Durham, NH 03824 USA

**Keywords:** *Serratia*, *Caenorhabditis*, Entomopathogen, EPN, Mutualism, Urea pathway

## Abstract

**Background:**

Entomopathogenic associations between nematodes in the genera *Steinernema* and *Heterorhabdus* with their cognate bacteria from the bacterial genera *Xenorhabdus* and *Photorhabdus*, respectively, are extensively studied for their potential as biological control agents against invasive insect species. These two highly coevolved associations were results of convergent evolution. Given the natural abundance of bacteria, nematodes and insects, it is surprising that only these two associations with no intermediate forms are widely studied in the entomopathogenic context. Discovering analogous systems involving novel bacterial and nematode species would shed light on the evolutionary processes involved in the transition from free living organisms to obligatory partners in entomopathogenicity.

**Results:**

We report the complete genome sequence of a new member of the enterobacterial genus *Serratia* that forms a putative entomopathogenic complex with *Caenorhabditis briggsae*. Analysis of the 5.04 MB chromosomal genome predicts 4599 protein coding genes, seven sets of ribosomal RNA genes, 84 tRNA genes and a 64.8 KB plasmid encoding 74 genes. Comparative genomic analysis with three of the previously sequenced *Serratia* species, *S. marcescens* DB11 and *S. proteamaculans* 568, and *Serratia* sp. *AS12*, revealed that these four representatives of the genus share a core set of ~3100 genes and extensive structural conservation. The newly identified species shares a more recent common ancestor with *S. marcescens* with 99 % sequence identity in rDNA sequence and orthology across 85.6 % of predicted genes. Of the 39 genes/operons implicated in the virulence, symbiosis, recolonization, immune evasion and bioconversion, 21 (53.8 %) were present in *Serratia* while 33 (84.6 %) and 35 (89 %) were present in *Xenorhabdus* and *Photorhabdus* EPN bacteria respectively.

**Conclusion:**

The majority of unique sequences in *Serratia* sp. SCBI (South African *Caenorhabditis briggsae* Isolate) are found in ~29 genomic islands of 5 to 65 genes and are enriched in putative functions that are biologically relevant to an entomopathogenic lifestyle, including non-ribosomal peptide synthetases, bacteriocins, fimbrial biogenesis, ushering proteins, toxins, secondary metabolite secretion and multiple drug resistance/efflux systems. By revealing the early stages of adaptation to this lifestyle, the *Serratia* sp. SCBI genome underscores the fact that in EPN formation the composite end result – killing, bioconversion, cadaver protection and recolonization- can be achieved by dissimilar mechanisms. This genome sequence will enable further study of the evolution of entomopathogenic nematode-bacteria complexes.

**Electronic supplementary material:**

The online version of this article (doi:10.1186/s12864-015-1697-8) contains supplementary material, which is available to authorized users.

## Background

We present the complete genome sequence and analysis of a novel *Serratia* species that, in conjunction with the nematode *Caenorhabditis briggsae*, forms a putative entomopathogenic association lethal to *Galleria mellonella* larvae [2]. This *Serratia* species was isolated from *C. briggsae* nematodes recovered from three separate *Galleria* traps baited in soil in the Kawa Zulu Natal province in South Africa and resembles the other entomopathogenic nematode (EPN) associations. EPNs are mutualistic associations between a bacterium and a nematode that enables them to kill insects and benefit both partners with nutrients and breeding sites [16, 41]. Although all three players in the EPN life cycle - pathogenic bacteria, nematode and host insect larvae – are ancient and abundant taxa in nature, only two independently evolved entomopathogenic partnerships are well studied. One is the association between bacteria in the genus *Photorhabdus* and Heterorhabditid nematodes [29, 100] and the other is the association between bacteria in the genus *Xenorhabdus* with Steinernematid nematodes [46]. EPN associations involve complex interactions between the pathogens and the nematode worms. In typical EPN associations the nematode is responsible for locating suitable host, penetrating the host insect and releasing the bacteria into the hemocoel while the bacteria are responsible for killing the host, bioconversion of complex compounds and protection of the insect cadaver from scavenging competitors thus ensuring nutrition for itself and its nematode partner [29, 100].

Significant bacterial adaptations to the EPN lifestyle include the regulation of the switch between mutualism and pathogenicity, accelerated insect killing, cadaver bioconversion, and re-association with infective juveniles [23, 48]. Recent studies have revealed that in both canonical EPN bacterial species L-proline in the insect hemolymph is the main trigger that initiates a metabolic shift from a quasi-dormant state in the nematode gut to a dramatic increase in secondary metabolite production in the insect hemocoel [33]. Following this L-proline-induced metabolic shift, major regulatory events take place. In the *Photorhabdus*/*Heterorhabdus* association, two global regulators, HexA [58] and Ner [69], control the switch between mutualism and pathogenesis, while the *phoP*/*phoQ* and the *astS*/*astR* two-component systems [38, 39] and the *pgbPE* operon [12] regulate pathogenicity and mutualism genes. Furthermore, Heterorhabditid nematodes fail to grow and reproduce normally when grown with *Photorhabdus* mutants defective in *ngrA*, suggesting that its phosphopantetheinyl (Ppant) transferase product is required for nematode growth and reproduction [27]. Finally, the reassociation of infective juveniles and their cognate bacteria as well as the retention of the bacteria in the nematode gut seems to be mediated by the expression of adhesion fimbriae encoded in one of the many genomic islands rich in phage remnants [46, 68, 86]. By comparison, in the *Xenorhabdus*/*Steinernema* association, a similar, but non-homologous, mechanism operates in which the global regulator Lrp and the two component system *cpxRA* and the *lysR* regulator *lrhA* [32] orchestrate all three major stages of the life cycle: infection, reproduction and transmission. Whereas many compounds are implicated in insect killing and sanitization of the insect cadaver [84, 85] transmission in *X. nematophila* seems to require the *nilABC* operon which encodes three surface-localized colonization factors whose mutations invariantly lead to defective recolonization of Steinernematid worms by *X. nematophila* bacteria [77, 78]. The absence of the *nilABC* genes in *X. bovienii* and in *Photorhabdus* species suggest that bacteria-nematode recolonization is realized by different mechanisms in these two well-studied EPN systems. To summarize, the genetic mechanisms by which *Photorhabdus* and *Xenorhabdus* achieve entomopathogenicity are quite distinct and evidence of independent evolution of a similar phenotype.

The bacterium we describe here, *Serratia* sp. SCBI, belongs to the genus *Serratia* that consists of several species with diverse lifestyles that include free soil dwellers [49], plant associates in the rhizosphere [11, 34, 88, 98], opportunistic pathogens [66, 96, 97] and obligate intracellular endosymbionts [22]. Most *Serratia* spp. secrete an array of active extracellular enzymes such as nucleases, proteases [17, 25], lipases [64] and hemolysin and have swarming and swimming mobility [4, 65, 70]. These features may enable them to colonize a wide variety of niches and contribute to their success as opportunistic pathogens. Of the sequenced *Serratia* spp., the closest species to our isolate, *S. marcescens* DB11, is a confirmed pathogen of invertebrates [49, 63], a function that is likely required for evolution of an EPN complex.

Although *Serratia* sp. SCBI was initially isolated as an associate of *Caenorhabditis briggsae*, this bacterium will also associate with strains of the well-studied model eukaryote *Caenorhabditis elegans* and allow these bacteriovorus nematodes to kill insects [2]. Both *C. briggsae* and C. *elegans* are well known associates of invertebrates [79, 83], a likely pre-adaptation to an EPN life cycle. Recent discoveries of *Serratia* spp. in EPN associations [89, 105] suggest the possibility that *Serratia* sp. SCBI may belong to a unique lineage within the genus *Serratia* that has evolved the capacity to confer an EPN lifestyle to diverse insect-associated nematodes. The *Serratia* sp. SCBI and Caenorhabditid EPN complex represents a unique opportunity to explore the evolution of symbiosis in a third EPN lineage. Here we present the complete genome sequence of *Serratia* sp. SCBI and explore how it is unique with respect to other closely related *Serratia* and how the predicted functional proteome compares to features found in the well-studied EPN-associated bacteria, *Xenorhabdus* and *Photorhabdus*.

## Results and discussion

### Overview of the *Serratia* sp. SCBI Genome Structure and Annotation

**Fig. 1:**
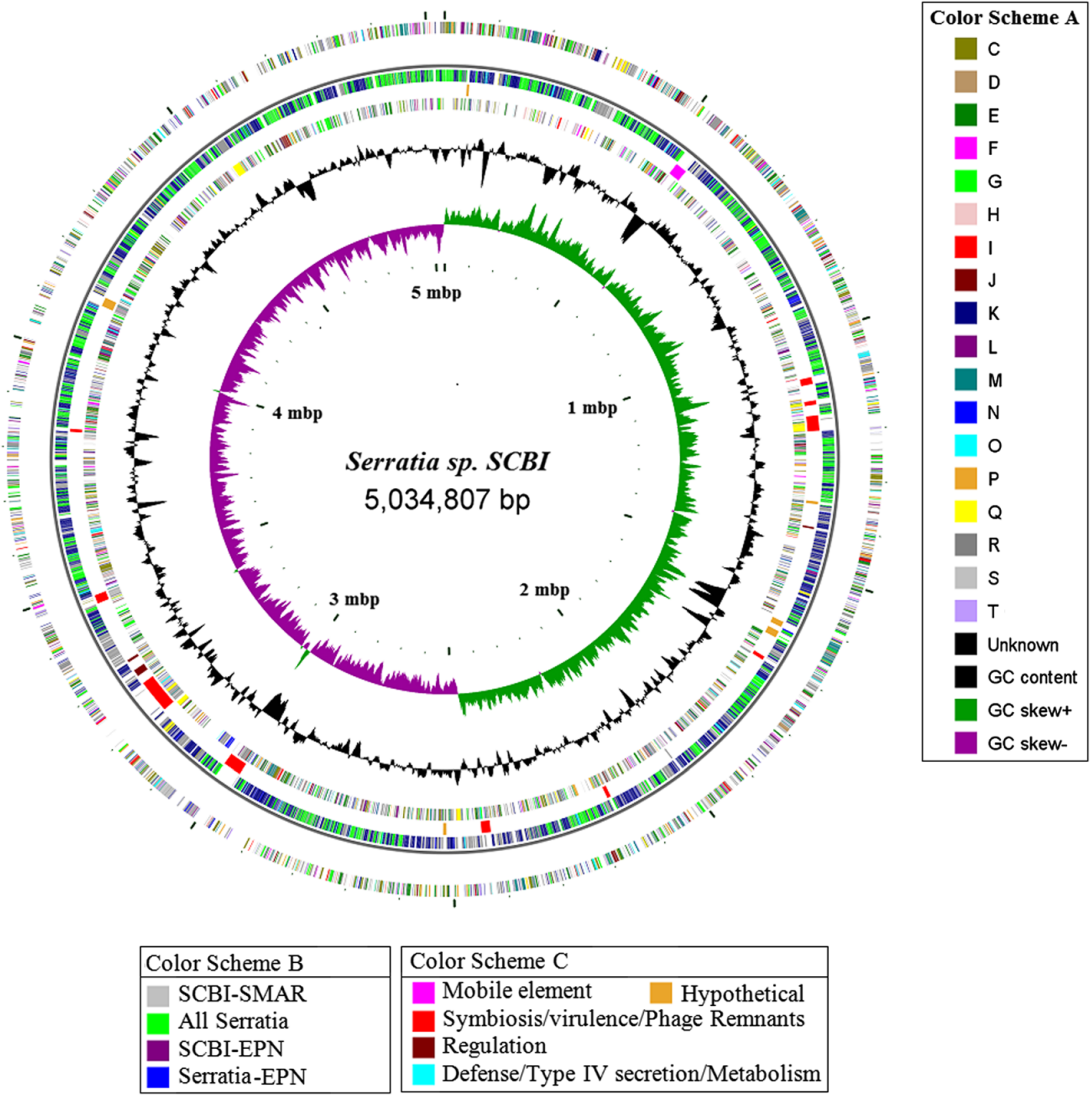
Circular representation of the *Serratia* sp. SCBI genome. Circular representation of Genomic features in *Serratia* sp. SCBI. From outer to innermost: First and fourth circles, genes in the plus and minus strands, respectively, by COG category (COG category color Scheme A, side panel); second circle, genes shared with other *Serratia* and EPN species (see color scheme B); third circle, genomic Islands (GIs) (Color Scheme C); fifth circle, GC content, sixth, innermost, circle, GC skew (Color Scheme A Side panel)

The genome of *Serratia* sp. SCBI is comprised of a single circular 5.04 Mb chromosome with an overall GC content of 59.7 %, 4599 predicted protein coding genes, 84 tRNA genes and seven sets of rRNA genes (Table 1, Fig. 1). In addition, *Serratia* sp. SCBI contains a single 64.8 Kb conjugative plasmid with 74 putative protein coding genes, 28 of which lack similarity to known proteins. (Additional file 1: Table S1: Plasmid ORFs). Blast analysis (data not shown) has shown that the *Serratia* sp. SCBI plasmid shows no obvious homology to the other known *Serratia* plasmid found in *S. proteamaculans* 568. The annotated genome is available at NCBI under accession numbers CP003424 and CP003425.

**Table 1:** Comparison of physical parameters of Serratia sp. SCBI with three of the sequenced Serratia genomes

Genome feature	Organism			
	S_SCBI	S_DB11†	S_AS12	S_568‡
Genome size (MB)	5.04	5.12	5.44	5.45
GC content (%)	59.3	59.1	55.9	55
Predicted ORFs	4599	4736	4952	4891
Protein coding DNA (KB)	4.4	4.49	4.73	4.75
Protein coding DNA GC content (%)	61.1	60.93	57.3	56.35
Genes with assigned functions	3736	3804	3994	4111
Genes without assigned functions	863	932	958	780
rRNA sets	7	7	7	7
tRNA genes	84	88	87	85
Plasmid Size(KB)	64.8	N/A	N/A	46.8
Plasmid GC content (%)	54.92	N/A	N/A	49
Plasmid encoded ORFs	74	N/A	N/A	51

*SMAR gene prediction: 4736 ORF by FgeneSB, 4763 SANGER prediction

† EBI, Sanger; ‡DOE, JGI

*S_SCBI Serratia* sp. SCBI; *S_DB11 S. marcescens* DB11; *S_AS12 Serratia* sp. AS12; *S_568 S. proteamaculans* 568

**Fig. 2:**
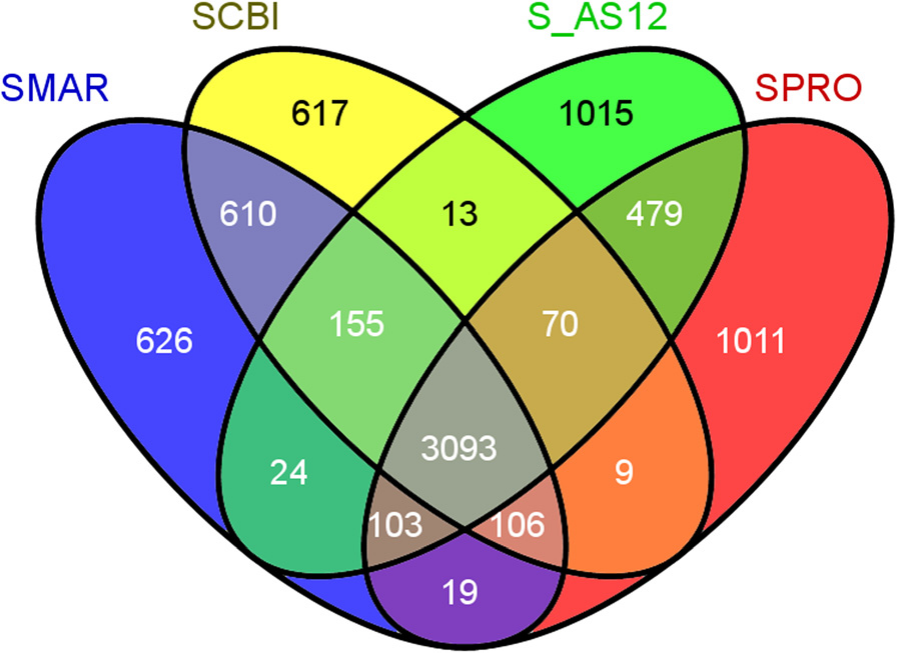
Venn diagram of shared and unique genes found in four *Serratia* genomes. The unique and shared genome among the compared genomes was determined by a dual cutoff of 30 % or greater amino acid identity and sequence length coverage of at least 70 %. Analysis was done using the MAUVE genome alignment tool [42]. SCBI: *Serratia* sp. SCBI; SMAR: *S. marcescens*DB11; SPRO: *S. proteamaculans*568, SAS12, *Serratia* sp. AS12

**Fig. 3:**
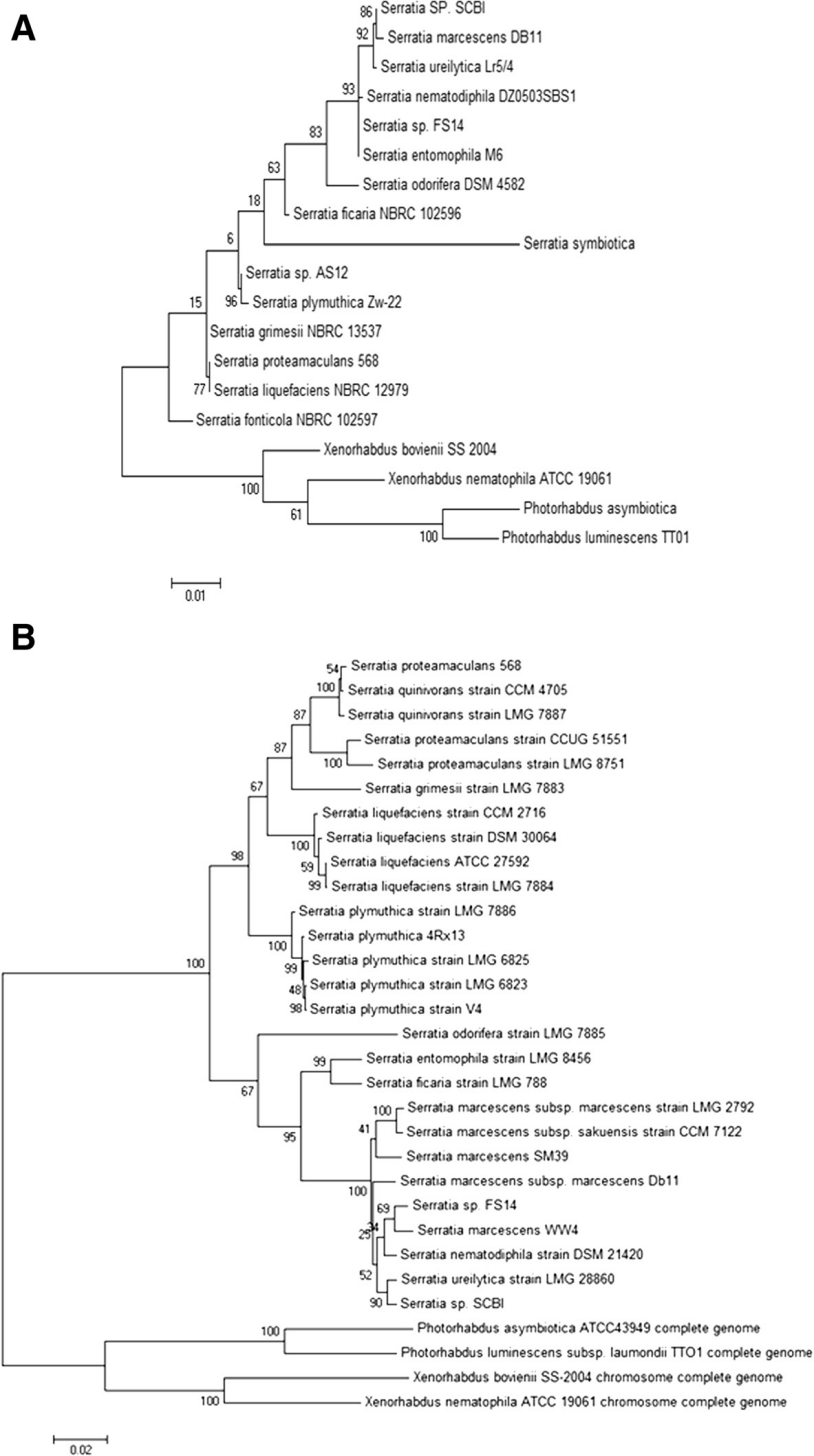
Evolutionary relationships of *Serratia* and representative bacteria from the entomopathogenic genera *Photorhabdus* and *Xenorhabdus.* Evolutionary relationships of *Serratia* and representative bacteria from the entomopathogenic genera *Photorhabdus* and *Xenorhabdus.* **a** Phylogenetic relationships inferred from the alignment of 1500 bp of 16S rDNA using the Maximum Likelihood [90]; **b** Phylogenetic relationships inferred from the alignment of 2623 bp of concatenated DNA from four housekeeping genes: *atpD* (634 bp), *gyrB* (742 bp), *ifnB* (613 bp) and *rpoB* (634 bp) using the Maximum Likelihood [90]. Numbers on internal branches are the results of Bootstrap analysis where the test was done with 1000 replicates [43]. Where applicable the trees are drawn to scale, with branch lengths in the same units as those of the evolutionary distances used to infer the phylogenetic tree. Evolutionary analyses were conducted in MEGA6 [91, 92]

Among complete *Serratia* genomes, the SCBI genome was the smallest in size and encoded fewer genes (Table 1). All the compared *Serratia* genomes shared a highly conserved genomic architecture as inferred from synteny of protein coding orthologs, tRNA genes, rRNA modules and their origins of replication. These genomes shared 3094 genes by MAUVE progressive alignment [35, 36] at 70 % coverage and 30 % identity. An additional 809 protein coding genes were shared by *S. marcescens* DB11 and *Serratia* sp. SCBI (Fig. 2). The *Serratia* sp. SCBI, *S. marcescens* DB11, *Serratia* sp. AS12 and *S. proteamaculans* 568 genomes had 519, 587, 1015 and 1011 unique genes, respectively (Fig. 2). An additional 1267 genes were shared between two or three species. The patterns of shared and unique genes are consistent with the evolutionary history of the species defined by 16S rDNA phylogeny (Fig. 3).

### Phylogenetic placement of SCBI

To place *Serratia* sp. SCBI in an evolutionary framework we compared the near complete 16S rRNA genes from diverse *Serratia* species and other relevant entomopathogenic bacteria (Fig. 3) as well as concatenated sequences from four housekeeping genes: *atpD, gyrB, ifnB* and *rpoB*. The phylogenetic tree from the concatenated housekeeping genes suggest that *Serratia* sp. SCBI is found in a clade of five *Serratia* which are all known insect associates and SCBI was closest to *S. ureilytica*. Among the well-studied and completely sequenced species included in the 16S phylogeny, *Serratia* sp. SCBI was most closely related to *S. marcescens* DB11, which is an insect pathogen [49, 50, 63] and antagonist of *C. elegans* [61, 76, 82]. This close relationship provides an ideal opportunity to identify candidate genes critical to the evolution of the EPN lifestyle. Another closely related species is *S. nematodiphila*, which was described as part of an EPN association with a Rhabditid nematode [104, 105]. Other close relatives include *S. entomophila* [50], which is pathogenic to New Zealand grass grubs, and *S. ureilytica* which is a nickel resistant *Serratia* isolated from the River Torsa in West Bengal, India [13]. This phylogeny suggests that *Serratia* sp. SCBI may represent a novel, independently evolving EPN association from within a lineage of *Serratia* commonly associated with insects.

### Genomic alignment and comparative genomic analysis

**Fig. 4:**
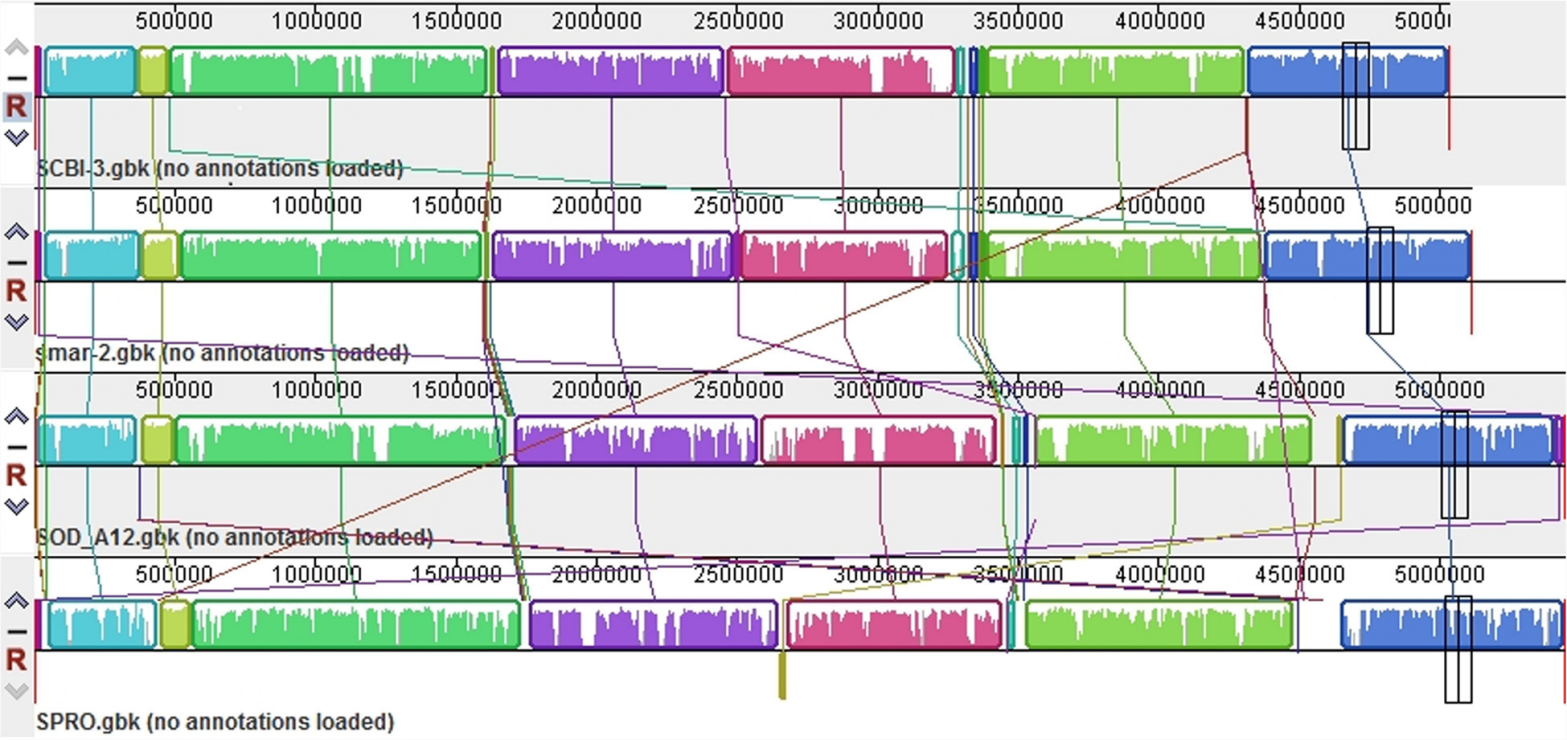
Genomic alignment of the four compared *Serratia* spp. Alignment statistics were generated and rendered by MAUVE progressive alignment software [35, 36]. SMAR: *S. marcescens* DB11, SCBI: *Serratia* sp. SCBI, and SPRO: *S. proteamaculans*586, SAS12: *Serratia* sp. AS12. Color schemes represent blocks of contiguous genes interrupted by colorless patches where the genomes differ from each other significantly and identified as GIs are located

Whole genome alignment of SCBI with three of the sequenced *Serratia* genomes show extensive synteny (Fig. 4) where large colinear blocks of genes are interrupted by insertions that resulted in genomic islands, deletions relative to SCBI and a small number of rearrangements involving relatively small genomic regions. In the context of this study, a genomic island (GI) was broadly defined as any stretch of five or more consecutive protein coding genes that: 1) are not present in the other members of the genus, and 2) show a divergent %GC content compared to the core genome. Our analysis predicts at least 29 GIs in *Serratia* sp. SCBI. Based on these criteria 326 of 519 (62.8 %) of unique genes are located in the genomic islands of SCBI and as discussed below most of these GIs encode functions of potential biological relevance to the EPN lifestyle (Table 2). To ascertain the significance of the *Serratia* sp. SCBI-*C. briggsae* association in relation to other *Serratia* and the established EPN bacteria, we conducted comparative analysis of the predicted proteome against the respective groups using the MAUVE [35] genome alignment tool. The cutoff values of 60 % identity and 70 % coverage, i.e. similarity of 60 % of the compared residues covering 70 % of the total length of the shorter sequence in the comparison, were used to include only hits with high confidence levels and exclude spurious hits (Table 3).

**Table 2:** Serratia sp. SCBI Genomic Islands and their putative/predicted phenotypes

GI#	Locus tags	Genomic location	Putative/Predicted role
GI-1	SCBI_0047-0052	46958..52205	Unknown
GI-2	SCBI_0298-0302	358841..362952	Adhesion
GI-3	SCBI_0465-0482	533080..558686	Unknown
GI-4	SCBI_0809-00817	928240..935735	Symbiosis/Metabolic
GI-5	SCBI_0963-0981	1080968..1096217	Virulence
GI-6	SCBI_1013-1019	1130653..1141194	Virulence, Defense
GI-7	SCBI_1042-1061	1164353..1197133	Virulence, Symbiosis
GI-8	SCBI_1205-1209	1349465..1355183	Defense
GI-9	SCBI_1258-1263	1405041..1410636	Symbiosis
GI-10	SCBI_1467-1478	1614753..1632962	Metabolic
GI-11	SCBI_1483-1498	1639797..1656736	Metabolic
GI-12	SCBI_1540-1547	1701960..1709609	Unknown
GI-13	SCBI_1814-1819	1993067..1996355	Unknown
GI-14	SCBI_1883-1888	2062882..2068451	Defense
GI-15	SCBI_1970-1975	2150551..2156393	Symbiosis
GI-16	SCBI_2176-2180	2367813..2374480	Unknown
GI-17	SCBI_2225-2232	2418731..2436006	Symbiosis
GI-18	SCBI_2295-2300	2513930..2520831	Metabolic
GI-19	SCBI_2738-2794	2980185..3019378	Defense, virulence, Adhesion
GI-20	SCBI_2953-3011	3191699..3264229	Defense, virulence, Adhesion
GI-21	SCBI_3036-3041	3289235..3297687	Metabolic
GI-22	SCBI_3184-3203	3460520..3479027	Adhesion, Defense
GI-23	SCBI_3369-3373	3652152..3658481	Symbiosis
GI-24	SCBI_3376-3380	3662181..3666951	Unknown
GI-25	SCBI_3539-3539	3835598..3842153	Defense
GI-26	SCBI_3778-3794	4112710..4132272	Defense, Symbiosis
GI-27	SCBI_3962-3966	4314901..4319983	Metabolic
GI-28	SCBI_4358-4366	4751221..4757933	Metabolic
GI-29	SCBI_4408-4412	4806030..4811621	Unknown

(see Additional file 2: Table S2 for the full/expanded version)

**Table 3:** The distribution of Virulence/defense/symbiosis factors in Serratia and the EPN bacteria

	SCBI	SMAR	SODO	SPRO	PASY	PLUM	XBOV	XNEM
Toxicity/Virulence								
GroEL	1	1	1	1	1	1	1	
Hemolysin	6	6	6	6	6	8	5	5
LopT					1	2		
Mcf					1	2	1	1
MtpB					1	3	1	1
MtpD					1	2	1	1
MtpE					1	2	1	1
NRPS-PKS	17	16	13	12	42	37	33	46
PirA	1	1	1	1	1	2		1
PirB-JHE					1	2		1
RtxA					3	4	1	1
TC				1	11	19	7	12
Symbiosis/Recolonization								
CipA					2	2		
CipB					1	1		
ExbD	2	2	2	2	2	2	2	2
HexA					1	1	1	1
LrhA	1	1	1	1				
NgrA	1	1	2	1	1	1	1	1
NilA								1
NilB								1
NilC								1
NilR					1	1	2	2
PbgPE	6	6	6	6	7	7	6	7
PhoP	2	2	2	2	1	1	1	1
PhoQ	1	1	1	1	1	1	1	1
Immune_evasion/Bioconversion								
Bacillolysin	1	1	1	1	1	1	1	2
Cif					1	1		
CpxA	1	1	1	1	1	1	1	1
CpxR	2	2	2	2	1	1	1	1
FlhC	1	1	1	1	1	1	1	1
FlhD	1	1	1	1	1	1	1	1
FliA	1	1	1	1	1	1	1	1
LPS	2	1	1	2	1	1	1	1
Lrp	2	2	2	2	2	2	1	1
MalP	2	2	2	2	1	1	1	1
MalQ	1	1	1	1	1	1	1	1
MalT	1	1	1	1	1	1	1	1
SctC					1	1		
Serralysin	4	4	2	2	1	1	1	1
XlpA					1	1	1	1

*SCBI Serratia* sp. SCBI; *SMAR S. marcescens* DB11; S_AS12, *Serratia* sp. AS12; *SPRO S. proteamaculans* 568; *PASY P. asymbiotica*; *Plum P. luminescens*; *Xbov X. bovienii*; Xnem, *X. nematophila*

### COG analysis among *Serratia*

**Fig. 5:**
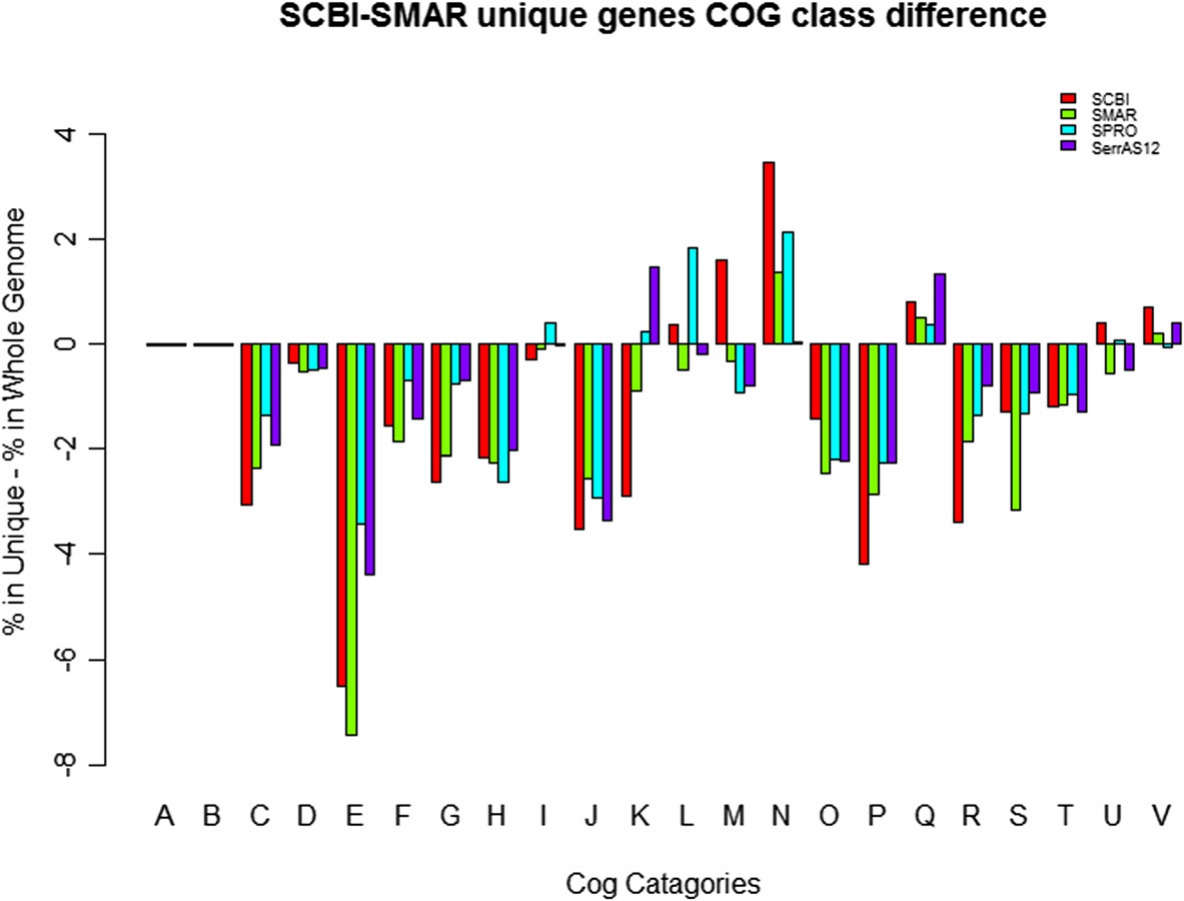
Relative COG category abundance in the core proteome in comparison with unique proteins in *Serratia*. The relative abundance of COG categories between the core and unique gene pools was calculated as follows: the number of proteins in each COG category was determined and the unique pools normalized to their respective total predicted protein numbers. Then the percentage of each COG category in the core proteome was subtracted from the corresponding COG percentage in the unique category and the difference plotted. COG functional categories descriptions are: [A] RNA processing and modification; [B] Chromatin structure and dynamics; [C] Energy production and conversion; [D] Cell cycle control and mitosis; [E] Amino acid metabolism and transport; [F] Nucleotide metabolism and transport; [G] Carbohydrate metabolism and transport; [H] Coenzyme metabolism; [I] Lipid metabolism; [J] Translation; [K] Transcription; [L] Replication and repair; [M] Cell wall/membrane/envelope biogenesis; [N] Cell motility; [O] Post-translational modification] protein turnover] chaperone functions; [P] Inorganic ion transport and metabolism; [Q] Secondary metabolites biosynthesis, transport and catabolism; [T] Signal transduction; [U] Intracellular trafficking and secretion; [R] General functional prediction only ; [S] Function unknown; [V] Defense mechanisms. [X] No cog category

Analysis of the functional categories and genome wide distribution of all unique genes with assigned Clusters of Orthologous Groups (COG) functions among the sequenced *Serratia* genomes revealed that the unique genes in *Serratia* sp. SCBI were biased towards categories that have direct bearing on the symbiosis and/or pathogenesis life style (Fig. 5). Specifically, the gains were in COG categories: [M]- Cell wall/membrane/envelope biogenesis; [N]- Cell motility; [Q]- Secondary metabolites biosynthesis, transport and catabolism; [U] - Intracellular trafficking and secretion and [V]- Defense mechanisms.

### Functional properties of *Serratia* sp. SCBI shared with other entomopathogens

To evaluate the *Serratia* sp. SCBI in the context of its role in an EPN complex, we searched the SCBI genome for homologs or functional analogs of all genes previously shown or implicated to be involved in host immune defense, host killing and cadaver protection, pathogenesis and symbiosis and reassociation in the canonical EPN species *Xenorhabdus* and *Photorhabdus* (Table 3). Although most of these genes appear in GIs in *Serratia* SCBI, we focused the comparison on three functions with well documented roles in EPN lifestyle but not confined to putative Genomic Islands: evading host defense, toxicity to host and competitors and recolonization. Functional studies on the *Serratia* sp. SCBI-*C. elegans* complex has shown that hemolysin, NRPS proteins (Petersen, LaCourse et al. submitted) and extracellular proteases are crucial to cytotoxicity and virulence in this putative EPN symbiosis [72]. The dynamics of mRNA expression of the alkaline metalloproteases (prtA1-prtA4) compared to the serine metalloproteases which peak before and after the death of the host, respectively, suggest a complex regulatory mechanism in killing the host and bioconversion of the cadaver [72]. Furthermore, inactivation of the hemolysin gene in *Serratia* sp. SCBI, which resulted in loss of hemolysis, failed to attenuate insecticidal activity but significantly increased motility and antimicrobial activity (Petersen et. al., submitted). Furthermore comparative study on the physiology of three sequenced strains, *Serratia* sp. SCBI, *S. marcescens* DB11 and *S. proteamaculans* 568, revealed that DB11 and SCBI were similar in insect virulence and cytotoxicity consistent with their phylogenetic proximity, but motility and lipase and hemolytic activities differed significantly between them [71]. The implication of these functional studies is that the *Serratia* sp. SCBI-*C. briggsae* association is a promising model to study the dynamics of gene expression during the transition from starved, inactive stay in nematode gut to active state during pathogenesis –killing and bioconversion– of insect hosts.

### Breaching host defense

Breaching the insect host’s defense –both the humoral and cellular components- is crucial for the establishment of infection. EPNs neutralize humoral response elements like lysozyme and cecropins with serralysin-like proteases such as the PrtA [42, 67] and by haemolysins (XhlA) and lipases (XlpA) and the FhlDC regulator operon [47, 67]. Cellular response evasion is directed mainly at suppressing the phenoloxidase pathway to prevent hemocyte aggregation and melanization. Several mechanisms are employed to suppress the insects’ cellular immune response. These include: inhibition of phospholipase A2 (PLA2) using stilbene and MalPQT operon products in *Photorhabdus* and expression of LPS in *Xenorhabdus,* the suppression of phagocytosis by Type III secretion system-mediated deposition of LopT and SctC directly into hemocytes [19, 20] and the production of cytotoxins such as Cif and MrxA that lead to apoptotic cell death. With the exception of the *xlpA* gene which is absent from *Serratia* spp. and the *cif* and *stc* genes that are absent not only from *Serratia* spp. but also from *Xenorhabdus* spp., all other genes identifies as instrumental in the toxicity of EPNS are present in *Serratia* spp. as well including SCBI. The presence of more serralysins in *Serratia spp.,* by comparison to the EPN genomes, suggests that the SCBI *C. briggsae* association may heavily rely on the expression of multiple serralysin genes and extracellular lipases and hemolysin production to breach the host immune system.

### Toxicity

Toxicity –both for killing the insect host and warding off bacterial and fungal competitors- is mediated by a plethora of insecticidal and bactericidal gene products [44]. These include the *Photorhabdus* Toxin Complex (TC) operons *tcaABCD* and their counterparts in *Xenorhabdus xptABCD*, as well as other toxicity genes like the *pirAB, xaxB*, *sepA*, *groEL*, *lopT*, *mcf*, *mcf2*, *rtxA*, *mptBDE*. Various drug efflux systems and numerous nonribosomal peptide synthase (NRPS) and polyketide synthase (PKS) genes [15, 37, 45, 53, 55] are also part of the overall toxicity caused by EPN systems. The redundancy evident in the toxin gene repertoire of *Photorhabdus* and *Xenorhabdus* is astounding – at the time of its sequencing in 2003, *P. luminescens* was described as having more genes encoding toxins than any other genome sequenced to date [40], however, *Serratia sp.* SCBI also has over 200 genes encoding toxins, NRPS genes, multiple drug efflux systems and assorted virulence factors.

### Nematode recolonization

Nematode recolonization involves two key elements: acquisition of partner bacteria by horizontal transmission via endotokia matricida [28], in which eggs hatch within the sacrificial mother to gain access to bacteria before emerging from the maternal corpse and the retention of bacteria in the gut of the infective juvenile. The latter seems to be mediated by *ngrA* [27, 59], the Type I fimbriae *mrxA* [24] and at least in *X. nematophila*, by the products of the *nilABC* and *nilR* genes [30, 31, 52]. Both of these requirements seem to be met in SCBI since *Caenorhabditids*, when grown on SCBI, almost exclusively reproduce by endotokia matricida [1] and SCBI possess homologs of both genes implicated in recolonization. Furthermore, the absence of *the nilABC* genes of *X. nematophila* in *Xenorhabdus boveinii* as well as *Photorhabdus* spp. show that colonization, like other aspects of EPN biology, can be produced by different mechanisms in EPNs.

From this comparison three patterns emerge: 1) *Serratia* lack homologs of the toxicity genes *lopT, rtxA, prtA, pirB, cif, mcf*, and *mcf*2 and the *TC* operons but have substantial number of secreted proteases and lipases, and hemolysins and 2) the majority of the *Photorhabdus/Xenorhabdus* virulence, symbiosis and regulatory genes are equally represented in *Serratia* (Table 3), and 3) the presence of key genes is not uniform among the different species –*nilABC* absent in *Photorhabdus* spp. and c*if, stc, cipAB* and *lopT* missing from *Xenorhabdus* spp.-suggesting that there is no single possible path to pathogenicity, cadaver bioconversion, repelling of competitors and bacterial re-association.

### Unique genes and Genomic Islands (GI) of SCBI

Apart from those summarized in Table 3, many other genes with well-documented pathogenicity/symbiosis functions in other microbial systems are found in the genome of SCBI more than half of which are located on genomic islands. Of the 29 genomic islands identified in *Serratia* sp. SCBI genome comparison (Fig. 1, third circle, Additional file 2: Table S2), many were enriched in defense and virulence genes and in - phage remnants which, in many bacteria including those in EPN associations, have been diverted into novel toxin/virulence factor delivery vehicles [8, 21, 56, 62].

Perhaps the most notable feature among the genomic islands is the urea pathway genes located on GI-4. The *hoxN/hupN/nixA* family nickel/cobalt transporter and the urea metabolism pathway proteins found in GI-4, are absent from other members of the genus *Serratia* but interestingly present in all the EPN bacteria. The *hoxN* transporter is necessary for urea hydrolysis [60, 103] making it an integral part of the pathway. While there is no evidence so far of a direct role for urea metabolism in the EPN lifestyle, a potential role for these pathways can be exemplified by current research on diatoms where the urea pathway is a key to facilitating rapid recovery from prolonged nitrogen limitation followed by rapid growth under nutrient rich conditions [6]. The EPN life cycle has a similar pattern in that the symbionts that survive in nutrient-limited conditions while in transit to the next cycle of infection face a sudden abundance of organic compounds upon entering the insect hemocoel, thus requiring rapid transitioning from starved state to exponential growth of the bacteria. Furthermore, the regulation of the urea pathway in the diatoms has been linked to proline [5] which has also been identified as the cue for the transition from starved to metabolically active state in *Photorhabdus* and *Xenorhabdus*.

Among the unique genes of SCBI that are relevant to its association with *C. briggsae* as a putative EPN are the O-antigen biosynthesis protein(SCBI_1044), NRPS proteins(SCBI_1017, SCBI_1055-1059, SCBI_2978), colicins (SCBI_1883-1887), Type IV secretion systems (SCBI_2992, SCBI_2984, SCBI_2994), and iron acquisition proteins(SCBI_1973-1975). These genes have been shown to be required for both symbiotic and pathogenic properties [12, 101, 102] and may play a similar role in the *Serratia*-*Caenorhabditis* EPN association.

The list of genes unique to SCBI also include efflux system proteins (SCBI_1013, SCBI_1208) that are specific for macrolide- a class of antibiotics that inhibit the growth of bacteria- and the resistance-nodulation- division (RND) efflux proteins (SCBI_3788-3791) that are known to actively scavenge antimicrobial compounds released by competitors or the host immune system [3, 14, 73, 74, 87, 93, 94], a predicted HtpX protease, a heat shock/stress inducible membrane bound zinc metalloprotease [81], the DinI family protein also known as MsgA (macrophage survival protein) (SCBI_3186) and prophage lysozyme proteins (SCBI_2756-2758) an endolysin/autolysin system known to degrade the peptidoglycan structures in bacterial cell walls [18, 51, 99]; Microcin H47(SCBI_2968, SCBI_3536), a bactericidal peptide antibiotic related to the Colicin V family secretion protein [10] and associated secretion ATPase required for its export [95]; several fimbrial proteins with functional annotations ranging from pilus assembly to anchoring which, in light of recent findings that implicate *Photorhabdus* and *Xenorhabdus* adherence to the gut walls of their respective worm associates via fimbrial proteins, [46, 68, 86] and two homologs of the HigB toxin protein/HigA protein (antitoxin to HigB)( SCBI_0907-0908), which in *Vibrio*, have been shown to inhibit cell growth in *Escherichia coli* upon ectopic expression [26].

## Conclusions

EPN associations are complex tripartite interactions between bacterial pathogens, symbiotic entomophagous nematodes and susceptible insect/insect larval hosts. Thus far our understanding of the mechanisms of EPN associations is limited to two superficially similar and convergent systems. Here we report the complete genome sequence of the bacterium involved in a novel independently evolving putative EPN association between a species of *Serratia* and nematodes in the genus *Caenorhabditis*. This *Serratia* is most closely related to another recently discovered EPN association, *Serratia nematodiphila,* and the well-studied insect pathogen *Serratia marcescens* DB11. Based on a comparison to the two well studies EPN systems, the genome of *Serratia* sp. SCBI contains a large number of genes that are potential candidates for EPN adaptations. Among the most notable shared functions with other EPN associates are the O-antigen, the *syrP* protein, several non-ribosomal peptide synthetases, bacteriocins, many fimbrial biogenesis and ushering proteins, secondary metabolite and toxin secretion systems and multiple drug resistance/efflux systems. However, the SCBI genome carries neither the TC complexes nor the *mcf* genes, which implies its use of different mechanisms of insect killing. The presence of several ORFs encoding putative virulence factors in horizontally acquired genomic islands suggest that EPN associations can be established by dissimilar sets of mechanisms for killing, bioconversion, sanitization and colonization. The presence of these sets of genes in many bacteria further suggests that the major hurdle in EPN complex formation may be the initial development of co-tolerance between potential partners. This complete genome sequence of one of the partners in a nascent EPN association should enable future analysis of the *Serratia/Caenorhabditis* EPN complex.

## Methods

### Bacterial isolation and identification

The bacterium was isolated from a *Galleria mellonela* trap laid in soil in the Kawa Zulu Natal province of South Africa. The detailed procedure has been previously described elsewhere [2]. Following isolation bacterial identity was determined by 16S rDNA PCR which shows 99 % identity with *Serratia marcescens* rDNA sequences at NCBI (CP003959.1).

### Genomic DNA isolation

Genomic DNA (gDNA) was isolated from overnight cultures grown in LB medium [10 g Bacto-tryptone, 5 g Bacto-yeast, 5 g NaCL, H2 O to 1 liter, pH 7.5] (BD, Sparks, MD) using the Qiagen Genomic DNA isolation kit (Qiagen, Germantown, MD) and following the procedure outlined for bacterial gDNA isolation. Precipitated DNA was collected by spooling the DNA using flame-sterilized and cooled glass rod. The spooled gDNA was immediately transferred to a microcentrifuge tube containing 1.5 ml sterile, nuclease free water. The DNA was dissolved on a shaker at 55 °C for 2 h. The yield, purity, and length of the DNA was determined for 1-5 μl samples by electrophoresis on 0.8 % agarose gel and by spectrophotometry on a NanoDrop 1000 (Thermo Scientific, USA).

### Fosmid Library construction

The fosmid library was constructed from the genomic DNA (gDNA) using the CopyControl pCC1FOS™ Vector that contains both the *E. coli* F-factor single-copy origin of replication and the inducible high-copy *oriV* according to the manufacturer’s protocol (Epicentre, Madison, U.S.A.). Briefly, gDNA was mechanically sheared by passing through a narrow gage sterile syringe then it was separated by pulse field gel electrophoresis (PFGE) overnight. A gel slice was excised from the 36-40 KB size window and the DNA extracted by gel extraction. The gel extracted gDNA was subsequently ligated into the fosmid vector exactly as described in the Epicenter protocol. The ligated mixture was then packaged into lambda phages using MaxPlax Lambda Packaging Extracts (Epicentre, Madison, U.S.A.). The packaged library was then transduced into *E. coli* EPI300™ Plating Strain, and transformants were selected on LB agar supplemented with 34 mg/ml Chloramphenicol. The library clones were picked by the Genetix Q-bot colony picking robot (Genetix Ltd, UK) and inoculated into 384 well plates and allowed to grow for 24 h at 37oC in humidified chamber. Colonies were stored in -80oC freezers until they were retrieved for downstream processing. To isolate fosmid DNA, randomly selected individual clones were grown overnight in Chloramphenicol supplemented LB broth and plasmid copy number were amplified by adding 5ul induction solution and incubating further for 4 hours. Fosmids were isolated by alkaline lysis method using Qiagen plasmid isolation buffers. The presence of recombinant DNA in the isolated fosmids and the polymorphism of the insert DNA were evaluated by agarose gel electrophoresis of BamHI (NEB, USA) digestion of the purified plasmid DNA.

### PCR primer design and amplicon sequencing

The genome sequencing gaps were closed using PCR based amplicon sequencing. To this end batches of PCR primer were designed by the Primer3 software driven by an in-house Perl script. Primers were synthesized by IDG (IDG Inc., MA, USA) and they were used to both amplify and sequence the amplicons after cleaning the PCR product from any unused primers. PCR fragments were purified using magnetic beads and SPRI solution. The primers were designed to have a melting point of 60 °C or above for increased specificity and simultaneous amplification at a single annealing temperature. Cycling conditions were as follows: Initial denaturation of plasmid, 5 min at 95 °C; 30 cycles or denaturation, annealing and primer extension at 95 °C 30 s, 60 °C 30 s and 2 min at 72 °C, respectively; a final synthesis hold at 72 °C for 10 min and a 4 °C hold until reactions were removed from the iCycler PCR machine (Biorad Inc. CA, USA). Long PCR for amplification of 3-8 KB fragments were performed the same way except that the 10X buffer was supplemented with additional MgCl_2_ to bring the final concentration to 25 mM and the extension time was increased to 6 min. All PCR amplifications were done using Finnzymes PCR kit (NEB, MA, USA) and the 15 mM 10X buffer was used for fragments between 100 to 2000 bases long.

### Fosmid end sequencing and quality monitoring

DNA isolated from 209 plasmids was end sequenced using the pCC1FOS™ forward and reverse primers designed by Epicenter. Half of the clones were sent to a commercial sequencing facility (Genewiz Inc, NJ, USA) the other half were sequenced in house by the ABI 3130 genetic analyzer. Efficiency of Fosmid library construction and the quality of the library were monitored by selecting at random 209 clones and subjecting them to different analyses. A subset of these (46 clones) were tested by Bam HI digestion and, except for a failed plasmid isolation in one clone, all 45 (100 %) of them gave distinct digestion patterns and one common band at 8 KB, representing the plasmid backbone. BLAST results showed that except for 4 clones that failed to sequence, probably due to sample cross over contamination, all fosmid end sequences hit the assembled genome at 34 to 45 KB apart in the right orientation giving an overall high efficiency of library construction and quality.

### Genome sequencing by 454 technology and assembly of contigs

An aliquot of the *Serratia* sp. SCBI gDNA was sent to the genome sequencing center at the University of Indiana for genome sequencing by 454 pyrosequencing technology. Two plates of genomic sequencing and one plate of 2 KB paired end sequencing were done to generate the complete genomic sequence of one circular chromosome and one large, circular plasmid of 68 KB size. The 454 sequencing resulted in ten contigs ranging in size from 2 KB to 3.5 MB and harbored 236 gaps of ranging from 4 nucleotides to 2 kb in length which were closed by PCR amplicon sequencing as detailed in the PCR primer design and amplicon sequencing section. The genome sequencing output from the 2 plates of 454 genomic reads and one plate of 2 KB paired end sequencing yielded 40 and 21 MB data, giving an overall 12X coverage of the genome. The main chromosome was resolved in seven genomic regions separated by seven sets of rDNA assemblies of 5 KB length as outlined below.

### Sequence gap filling, genome assembly and bioinformatics analysis

The 10 scaffolds assembled by the Newbler Assembler sequence initially included 236 gaps ranging from 4 to 2050 bases long. PCR based amplicon end sequencing resolved all the gaps bringing the sequence assembly to a satisfactory level of completion. PCR primers were designed on regions flanking gaps by the Primer3 program [80] after orientation and order of scaffolds were determined by a combination of bioinformatics and laboratory based methods. Briefly, truncated scaffold sequences consisting of 500 bp from the start and end of each scaffold were generated by a Perl script. These truncated sequences were then blasted against the *S. marcescens* DB11 genome which identified their counterparts unambiguously showing a great deal of synteny between the two genomes. This finding was then confirmed by long PCR performed using primers pointing outward from the pairs of gap ends predicted by the bioinformatics analysis. All of the predicted combinations resulted in the amplification of the expected 6 KB fragment confirming both the order and orientation of the clones as well as their size. The remaining two fragments were resolved into a circular plasmid by blast hit with two fosmid clones which hit the two scaffolds unambiguously. This was also confirmed by PCR that amplified predicted size of amplicons from primers designed at the ends of the two scaffolds and individual fosmids used as template. Long PCR and amplicon sequencing were performed as described above and the resultant amplicon sequences were then added to the 454 reads and reassembled by the Newbler to generate the complete genome sequence. Comparative genomic analysis of *Serratia* sp. SCBI genome with the two other completed genomic sequences in the genus, *S. marcescens*DB11 and *S. proteamaculans*568 was done to determine the genes shared between the three genomes and the three way paired genomes (*Serratia* sp. SCBI-DB11, *Serratia* sp. SCBI-SPRO, DB11-SPRO) and the genes unique to each of them using MAUVE [35, 36], MURASAKI [75] and Blast [7, 57] bioinformatics tools, the RAST Annotation Server [9] and in house developed Perl scripts. A comparison was also done with *P. luminescens* and *X. nematophilia* to shed light on the entomopathogenic aspects of the *Serratia* sp. SCBI-*Caenorhabditis* relationship and to determine whether it resembles the *Photorhabdus*/*Heterorhabdus* or the *Xenorhabdus*/*Steinernema* type association or it constitutes a novel class of EPN association with a different entomopathogenic signature driven by a novel set of genes and pathways.

### Accession numbers

The genome sequence and its annotations are available at NCBI under the accession numbers CP003424 and CP003425.

## Additional files

Additional file 1: Table S1.
*Serratia* sp. SCBI predicted plasmid encoded genes. Additional file 2: Table S2.
*Serratia* sp. SCBI Genomic Island genes with putative/Predicted functions relevant to EPN life style.
